# Construction and evaluation of an alcohol vapor chamber system

**DOI:** 10.7555/JBR.36.20220151

**Published:** 2022-10-28

**Authors:** Wan Jiang, Jiajia Chen, Olivia Ewi Vidjro, Yingying Zhang, Gengni Guo, Ziyi Li, Yize Qi, Rouli Dai, Tengfei Ma

**Affiliations:** 1 Grade 2018, School of Pharmacy, Nanjing Medical University, Nanjing, Jiangsu 211166, China; 2 School of Pharmacy, Nanjing Medical University, Nanjing, Jiangsu 211166, China; 3 Translational Medicine Research Center for Drug Dependence and Withdrawal, Nanjing Medical University, Jiangsu 211166, China; 4 National Institute of Drug Clinical Trial, The Fourth Affiliated Hospital of Nanjing Medical University, Nanjing, Jiangsu 210031, China

**Keywords:** alcohol use disorders, alcohol vapor model, anxiety, depression, NMDA receptor

## Abstract

An increasing number of studies demonstrated that alcohol vapor chamber is an effective way to model physical signs of alcohol use disorders. Although researchers are developing different vapor chambers to study chronic alcohol exposure model worldwide, few studies build and modify their own vapor chambers in China. Here, we designed and established an alcohol vapor chamber system for small animals. We described a paradigm showing how to control and monitor alcohol concentration in whole system. The vapor chamber system with several advantages including accommodating up to ten standard mouse cages. Furthermore, the system was tested by evaluating the blood alcohol concentration and neuron injury in mice. Importantly, the alcohol withdrawal after vapor exposure caused motor coordination impairment, anxiolytic- and depression-like behavior. Finally, the N-methyl-D-aspartate receptor (NMDAR)-mediated glutamatergic transmissions in the medial prefrontal cortex was changed after alcohol vapor exposure-induced behaviors. The frequency and amplitude of spontaneous excitatory postsynaptic currents between control and alcohol groups were not different, suggesting that alcohol exposure-induced behaviors are associated with the change in NMDAR response. Taken together, the new alcohol vapor chamber system was constructed, which would help to research the relationship between the stable alcohol exposure and withdrawal behaviors and to study chronic alcohol exposure-induced disorders in China.

## Introduction

Alcohol use disorder is a medical condition caused by repeated excessive drinking or chronic alcohol consumption, eventually resulting in compulsive drinking or withdrawal reaction^[1]^. This process usually takes a long time and produces harm to humans both physically and mentally^[2]^. An increasing number of studies try to develop animal models to parallel the human condition^[3]^. The difficulty for animal model of alcoholism is how to efficiently cause excessive alcohol exposure that may lead to withdrawal-induced maladaptive behaviors. Therefore, the construction of animal models to induce high levels of alcohol consumption is essential to the research of alcohol use disorders and the identification of the therapeutic targets.

Alcohol vapor inhalation is one of forced administration of alcohol model, which can induce high levels of alcohol in the body^[4]^. In this model, the animals passively inhaled alcohol vapor by means of atomizing vapor^[5]^. The alcohol vapor is absorbed into the blood through the lungs of the animals, bypassing the initial metabolism of alcohol by the liver, and stabilizing the blood alcohol concentration (BAC) at a higher level. More alcohol enters the brain through the blood-brain barrier, resulting in faster achievement of BAC and more withdrawal symptoms^[6]^. Therefore, the rapid rise and stability of BAC induce clear signs of intoxication (excessive drinking in a short period). Unlike voluntary alcohol consumption with unstable BAC^[7]^, alcohol vapor inhalation makes experimental animals easily induce physical signs of chronic alcohol exposure in less time^[6,8]^. The model is established and the machines have been commercially manufactured. However, the existing types of machines still need improvement. More focus is required on variables such as maintaining a balanced pressure in the chamber to prevent animal death, maintaining a sufficient oxygen supply to keep the animals healthy, and controlling the concentration of alcohol vapor in the chamber. Taken together, the construction and investigation of alcohol vapor conditions are still in the exploratory stage and the manufacture of alcohol vapor devices needs to be established in China.

In this study, we designed a new alcohol vapor chamber system to establish a chronic alcohol exposure model in mice. This new system has several advantages. All the cages were placed in the same chamber. The large capacity exposure chamber combined with a multi-module and contained more numbers of modeling animals. The rate of air was regulated by one controller. An environment-monitoring module, an exhaust treatment device, and a multi-module function maintained an appropriate proportion of air and stable alcohol vapor concentration, which reduced animal mortality. The exploration of settings suggested that the constructed system successfully induces sufficient BAC in mice. More and more studies showed that chronic alcohol exposure induced anxiety and depression^[9]^. We used vapor exposure-induced motor coordination, anxiolytic- and depression-like behaviors to evaluate this model. Finally, the changed behaviors were associated with increasing N-methyl-D-aspartate receptor (NMDAR)-mediated glutamatergic transmissions after alcohol vapor exposure^[10]^. The device we established can expand the research on modeling conditions of alcohol exposure-induced behaviors, and provide high-quality animal models for related studies.

## Materials and methods

### Animals

Male C57BL/6 mice (8 weeks old, weighting 25 to 30 g) were purchased from Laboratory Animal Centre of Nanjing Medical University, China. Animals were housed individually at 25 ℃ under a 12-h light/dark cycle, with lights on at 7:00 AM. Food and water were provided *ad libitum*. All animal experiments were approved by the Ethics Committee of Animal Care of Nanjing Medical University (Approval No. IACUC-2105026) and followed the Guidelines for Animal Experiments of the Chinese Academy of Medical Sciences.

### Reagent

Isopropyl alcohol, aluminum potassium sulfate (analytically pure), and heparin sodium were purchased from Sinopharm Chemical Reagent Co., Ltd. (China). Nissl Staining Solution (Cresyl Violet) was purchased from Beijing Solarbio Science & Technology Co., Ltd. (China). Other reagents were obtained from MilliporeSigma (USA).

### Design and construct the alcohol vapor chamber system

**Figure 1:**
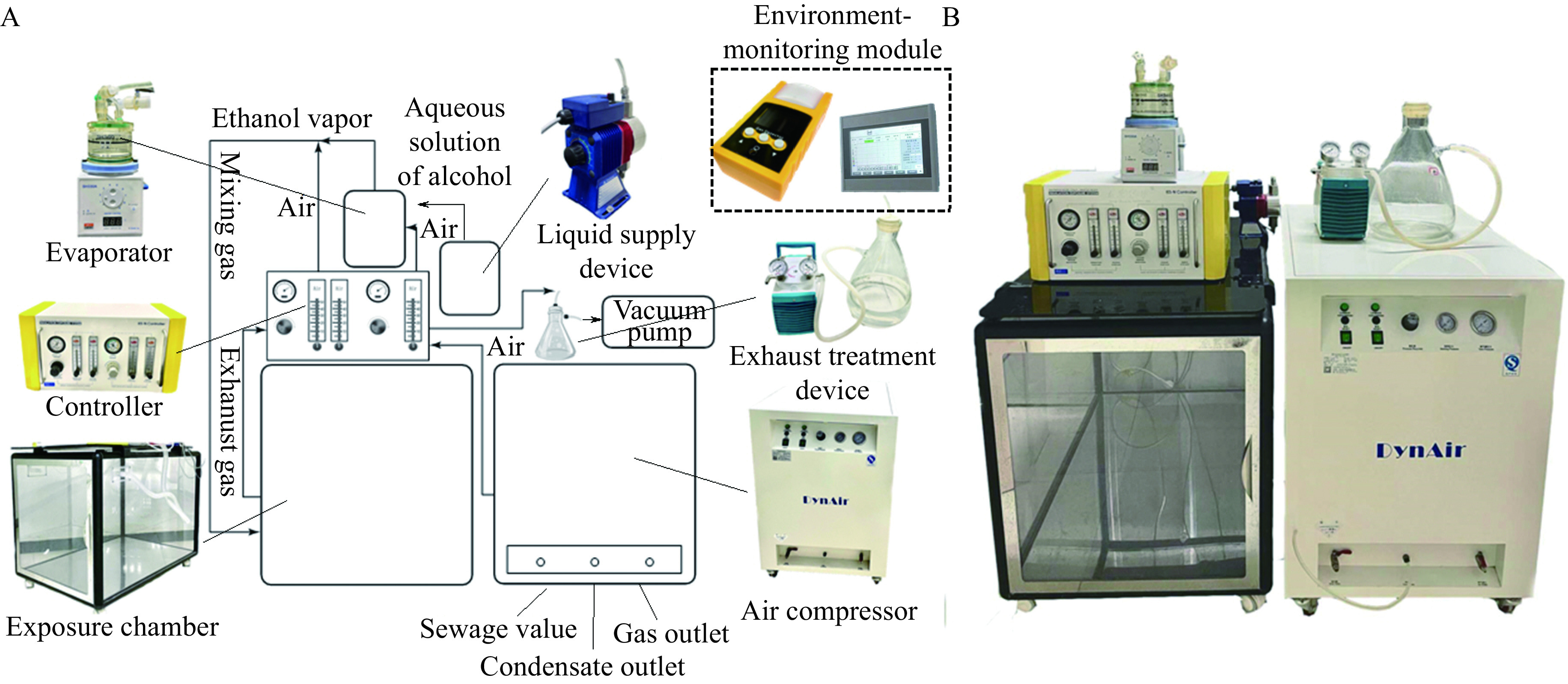
The design and construction of alcohol vapor chamber system.

The aim of designing the alcohol vapor chamber is to maintain the living conditions of animals and appropriate inhalation concentration of alcohol that does not cause animal death. The vapor chamber system consists of a vapor generation module, an environment-monitoring module, an exposure device, and an exhaust treatment device (***Fig. 1A*** and ***B***). a) The vapor generation module is further divided into an air compressor, alcohol evaporator, and liquid supply device (LSD). The air compressor pumps the appropriate ratio of air, together with alcohol vapor from the alcohol evaporator, into the exposure chamber through the air tube and dilution tubes. LSD provides the alcohol into the alcohol evaporator. The device keeps various gases in a relatively stable state in exposure chamber. The concentration of alcohol vapor meets the requirements of model establishment. b) The environment-monitoring module consists of a breathalyzer (Marcuss YC-16, China) for measuring alcohol concentration and a controller for monitoring the content of CO_2_, O_2_, and humidity in the chamber. c) The exposure device, which is a chamber, accommodates up to ten standard mouse cages. d) The exhaust treatment device deals with waste gas to protect the health of the laboratory staff and keeps the pressure of the chamber in balance. It is also minimized the impact on the environment. During the whole experiment, the mice with padding, food, and water bottles were put into the exposure chamber. After pumping alcohol to the evaporator, we controlled the pump force and frequency to keep the alcohol liquid level at a standard level. Then we used the controller to adjust the gas rate in air and dilution tubes. These methods will gradually increase the concentration of alcohol vapor to reach the range of 20 to 27 mg/L for about 10 min. By moderating related parameters, we controlled O_2_ concentration at the range of 19% to 21% and kept CO_2_ level below 0.3%. The real-time relative humidity was monitored and maintained at the range of 45% to 50% during exposure. To prevent experimental accidents, it is important to pay attention to maintaining the alcohol liquid level in the evaporator between the minimum and maximum range. The waste from the exhaust valve of the air pump should be cleaned up regularly (water vapor, dust, *etc.*). The exhaust absorption liquid should be replaced regularly to ensure the exhaust gas treatment efficiency. When closing the instrument, the air pump should be closed first, and then the exhaust treatment device and other components should be closed. This will ensure the air pressure is relatively stable in the exposure chamber and protect the health of experimental animals.

### Modeling machine and parameter debugging

The concentration of alcohol in the vapor chamber is controlled by adjusting parameters such as the temperature of the alcohol evaporator, the speed of air pump, and flow rate of the gas. It is also detected by a breathalyzer. When the concentration of alcohol vapor exceeds the maximum measurement range of the breathalyzer, a certain amount of gas is extracted from the exposure chamber, diluted in a ratio of 1:16, to decrease the concentration of alcohol vapor. To create different exposure room concentration gradients of alcohol vapor, the mice divided in groups A, B, and C were exposed to alcohol with 20%, 50%, and 95% alcohol for 4 h under the conditions of an alcohol evaporator at 25 ℃, air pump speed of 30 L/min, and an airflow rate of dilution tube of 10 L/min, respectively. The control group mice were exposed to air for 4 h under the same conditions. Considering the different humidity of the vapor environment formed by different concentrations of alcohol, we use silica gel to control the moisture. We defined the following parameters: a) Evaporation temperature of alcohol evaporator. By changing the temperature, the speed of alcohol evaporation can be reached the concentration of alcohol vapor in the exposure chamber. b) Flow rate of air pumped into the alcohol evaporator from the air pump. The faster the flow rate of air pumped into the air pump, the faster the evaporation rate of alcohol in the alcohol evaporator, and the higher the concentration of alcohol vapor in the exposure chamber. c) Air flow rate in the dilution tube. The alcohol vapor in the alcohol evaporator is mixed with the air transported in the dilution tube and passed into the exposure chamber. The faster the airflow rate in the dilution tube, the lower the concentration of alcohol vapor in the exposure chamber. d) Alcohol concentration. The concentration of alcohol vapor changes as the concentration of alcohol in the LSD changes. By adjusting the above parameters, the liquid level in the alcohol evaporator was controlled to remain relatively stable, so the concentration of alcohol vapor in the exposure chamber was controlled to remain relatively stable^[11]^.

### Chronic alcohol exposure

We used the above device for mouse alcohol exposure modeling. Mice in alcohol group were placed in an alcohol vapor chamber, treated with 95% alcohol, alcohol evaporator at 25 ℃, air pump rate of 30 L/min, and an airflow rate of 10 L/min in the dilution tube. The alcohol solution was made of water. Under these conditions, they were continuously exposed for 5 days a week for 4 h a day for 4 weeks. Mice in the control group were given air. The method was the same as that in alcohol group.

### Blood alcohol concentration detection

#### Solution preparation

Before determining plasma alcohol concentration, we prepared the following solutions. The concentration of isopropyl alcohol solution was 0.5 mg/mL. The concentration of alcohol standard solution was 2.0 mg/mL. It accurately placed 126.6 µL anhydrous alcohol in a 50 mL volumetric bottle, mixed with 25 mL double distilled water. Then different concentrations of alcohol standard liquid were prepared respectively. The protein precipitator was an aluminum potassium sulfate saturated solution.

#### Blood sample processing

We collected blood from the mice's orbit after they were exposed to different concentrations of alcohol in a vapor chamber. Eye blood (1 mL) from each group of mice was added to Eppendorf (Ep) tubes (MilliporeSigma) containing 50 μL of 100 mg/L sodium heparin solution, mixed well, and then centrifuged at 4 ℃ (13 934 *g*, 5 min). 0.125 mL of plasma from blood samples of control mice were added to Ep tubes containing 0.25 mL of isopropanol solution as a blank negative control, and 0.1 mL of plasma from blood samples of control mice were added to Ep tubes containing 0.25 mL of isopropanol solution and 0.025 mL of alcohol standard solution as a blank positive control. The samples were centrifuged again to precipitate and remove protein (13 934 *g*, 5 min)^[11]^.

#### Conditions of gas chromatographic

The blood alcohol concentrations were measured by using gas chromatography with a GC-2014 (Shimadzu, Japan). The gas chromatographic conditions are as follows: the temperature of column is 30 ℃, sampling chamber 150 ℃, and the detector 100 ℃; the velocity ratio of carrier gas (nitrogen) to hydrogen is 0.6:1; the volume of injection is 1.0 µL.

### Rotarod test

The rotarod test was used to evaluate motor coordination and reflect parkinsonian symptoms such as akinesia, and rigidity^[12]^. After exposure to air (control group) or 95% alcohol (alcohol group) and a withdrawal of 24 h, the two groups of mice were subjected to behavioral test. Mice were trained with rotation speed increasing from 5 to 40 rpm in 5 min. The time that the mouse remained on the rod was recorded in three trials and averaged^[13]^.

### Forced swim test

The transparent cylindrical glass container was 25 cm in height and had a diameter of 20 cm. We placed the mice in a container filled with water (25 ℃) for 6 min. Coded the duration of time spent as *'Immobile'* in the last 4 min if the mouse was floating without any movement except for those necessary for keeping the nose above water, gently dry the mice with paper towels before returning them to their cages. Analyze statistically the duration of time spent as *'Immobile'* within 4 min and evaluate the degree of depression of mice in each group^[14]^.

### Open field test

The open field consisted of a box (40 cm×40 cm×40 cm), and it was divided into a center zone (25% around the arena) and an outer zone in the periphery^[15]^. The mice were placed in the middle of the box and allowed to adapt to the new environment for a few minutes. We used Trackermaster software^[16]^ (Zongshi, China) to record the behavior of mice for 10 min and statistically analyzed the percentage of central time of mice. The box was cleaned with 75% alcohol and dried between each experiment to remove odor.

### Nissl staining

At the end of the behavioral tests, the control and alcohol group mice were sacrificed under anesthesia and perfused with phosphate buffer saline (pH 7.4) and 4% paraformaldehyde. After dehydration and cutting, the slice was blown dry and stained in 0.1% Cresyl Violet for 4 to 15 min, then were soaked in 70%, 95%, and 100% alcohol for 3 to 5 min. The neuropil would be stained a granular purple-blue^[17]^. The slices were fixed by immersion in xylene, sealed by glycerol, dried in a fume hood, and visualized by microscopy (Nikon, Japan). The minimal pixel was set at 50 pixels, and the average optical density values of Nissl bodies was analyzed. To calculate the relative optical density, the optical density of Nissl bodies in the alcohol group was divided by that of the control group.

### Electrophysiology

After exposure to air (control group) or 95% alcohol (alcohol group) and a withdrawal of 24 h for behavioral tests, the two groups of mice were sacrificed for electrophysiological measurements. Slice electrophysiology was performed as previously described. The coronal sections (250 µm) with the medial prefrontal cortex (mPFC) were prepared in an ice-cold cutting solution containing (in mmol/L): 40 NaCl, 148.5 sucrose, 4 KCl, 1.25 NaH_2_PO_4_, 25 NaHCO_3_, 0.5 CaCl_2_, 7 MgCl_2_, 10 glucose, 1 sodium ascorbate, 3 sodium pyruvate, and 3 myoinositol, saturated with 95% O_2_ and 5% CO_2_. Slices were then incubated in a 1:1 mixture of cutting solution and external solution at 32 ℃ for 45 min. The external solution contained the following (in mmol/L): 125 NaCl, 4.5 KCl, 2.5 CaCl_2_, 1.3 MgCl_2_, 1.25 NaH_2_PO_4_, 25 NaHCO_3_, 15 sucrose, and 15 glucose, saturated with 95% O_2_ and 5% CO_2_. Slices were then maintained in an external solution at room temperature until use.

Slices were perfused with the external solution at a flow rate of 3–4 mL/min at 32 ℃. Whole-cell patch-clamp recordings were made using a Sutter patch IPA-2 amplifier controlled by Igor pro 9 software (Sutter Instrument, USA). For whole-cell voltage-clamp recordings, we used a Cs-based solution, containing (in mmol/L): 119 CsMeSO_4_, 8 TEA.Cl, 15 HEPES, 0.6 EGTA, 0.3 Na3GTP, 4 MgATP, 5 QX-314.Cl, 7 phosphocreatine. The pH was adjusted to 7.3 with CsOH. Bipolar stimulating electrodes were positioned 100–150 µm away from the recording electrode used to record glutamatergic transmission in mPFC neurons. All measurements were conducted in the presence of the GABA_A_ receptor antagonist Bicuculline (10 µmol/L).

### Statistical analysis

SPSS 13.0 (International Business Machines Corporation, USA) was used as our data analysis tool. All data were expressed as mean±SEM. Statistical significance was assessed using the unpaired or paired *t*-test, One-way ANOVA, and two-way RM ANOVA followed by Student-Newman-Keuls (SNK) methods. Statistical significance was set at *P*<0.05.

## Results

### Evaluation of the parameters in the alcohol vapor chamber system

**Figure 2:**
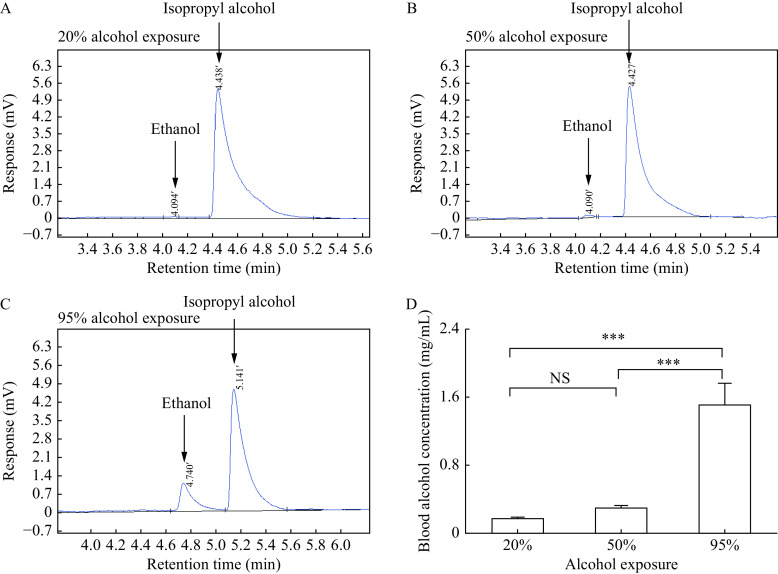
Evaluation of alcohol vapor chamber system by measuring blood alcohol concentration.

**Figure 3:**
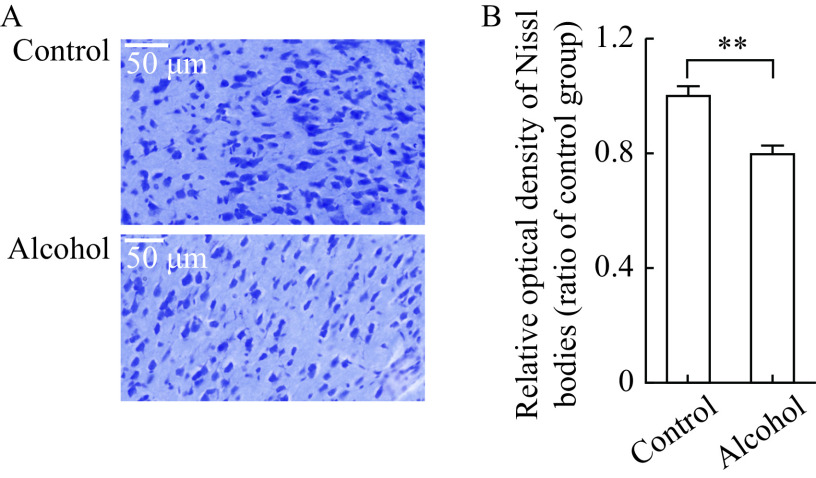
Evaluation of alcohol vapor chamber system by Nissl staining of mPFC neurons.

To evaluate the alcohol vapor chamber system, we measured the BAC of mice in different concentrations of alcohol exposure groups. Adding the standard ethanol solutions and isopropyl alcohol internal solutions in plasma of control group, using the mixed solutions to establish a standard curve. The corrected curve of BAC was calculated as y=0.1949x−0.0237, *R*²=0.9976. The y-axis stands for the BAC and the x-axis stands for the ratio of alcohol and isopropanol peak area. After exposure to 20%, 50%, and 95% alcohol for 4 h, the plasma alcohol concentration of mice was 0.17 mg/mL, 0.30 mg/mL, and 1.51 mg/mL, respectively (***Fig. 2A***–***D***). The BAC in 95% alcohol exposure group was significantly higher than that of in 20% and 50% alcohol groups, which can reach high level (1.5–2.0 mg/mL). Another evaluation was neuronal injury. The results showed that the number of Nissl bodies in mPFC of the alcohol group mice was less than that of the control group (*P*<0.01) (***Fig. 3A*** and ***B***). Taken together, we determined the final establishment conditions, suggesting that our constructed device is successful in inducing chronic alcohol exposure and injury model.

### Alcohol vapor exposure-induced the withdrawal behaviors

**Figure 4:**
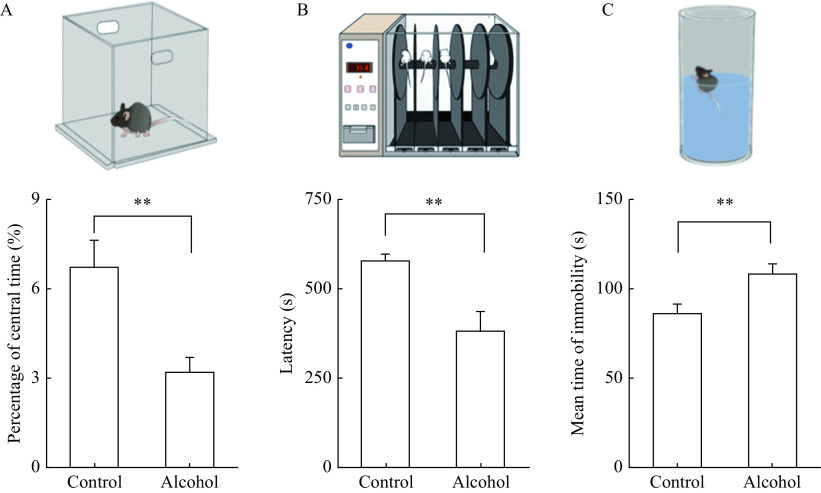
Changes of experimental device diagram and behavioral evaluation index of depression- and anxiolytic-like behaviors in mice.

To test a successful vapor model of alcohol-induced withdrawal behavior, mice were exposed to alcohol under the above modeling conditions. On the first day after 24 h of alcohol withdrawal, the mice were trained by the open field test. Compared with the control group, the time spent in the center of the open field of the alcohol-exposed mice was significantly decreased. The alcohol group mice were more inclined to stay in the surrounding area, and the degree of anxiety caused by the new open environment was significantly increased (***Fig. 4A***). Furthermore, the rod rotation experiment was carried out on the second day, we found that compared with the control group, the latency of mice to fall in the alcohol group was significantly shorter, indicating that the endurance and motor coordination of mice would be significantly weakened, and the difference was statistically significant (***Fig. 4B***). Taken together, the vapor chamber exposure induced signs of alcohol withdrawal suggesting that our constructed system is sufficient to induce alcohol-related behaviors. Lastly, we carried out the forced swimming test on the third day, the mean time of immobility of alcohol group mice in the last four minutes increased obviously compared with that of the control group mice (***Fig. 4C***). This suggests that mice in the alcohol group were more likely to be in a "state of despair" if they were confined to an unavoidable stressful situation. Therefore, the depression level of the model group was higher than that of the control group, and the difference was statistically significant.

### The glutamatergic transmission changed in mPFC neurons after alcohol vapor modeling

**Figure 5:**
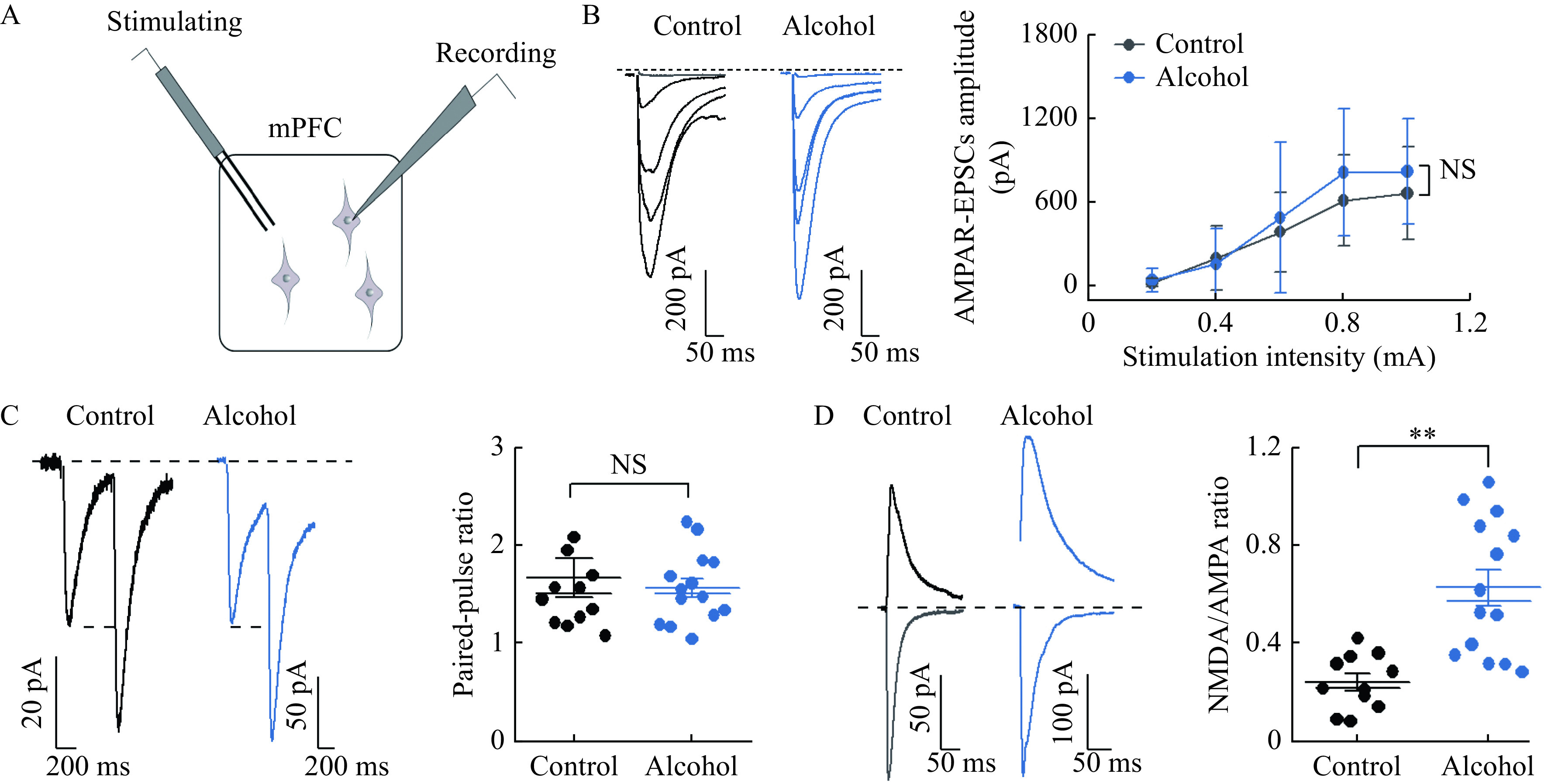
The NMDAR-, but not AMPAR-mediated glutamatergic transmission changed after vapor alcohol exposure.

**Figure 6:**
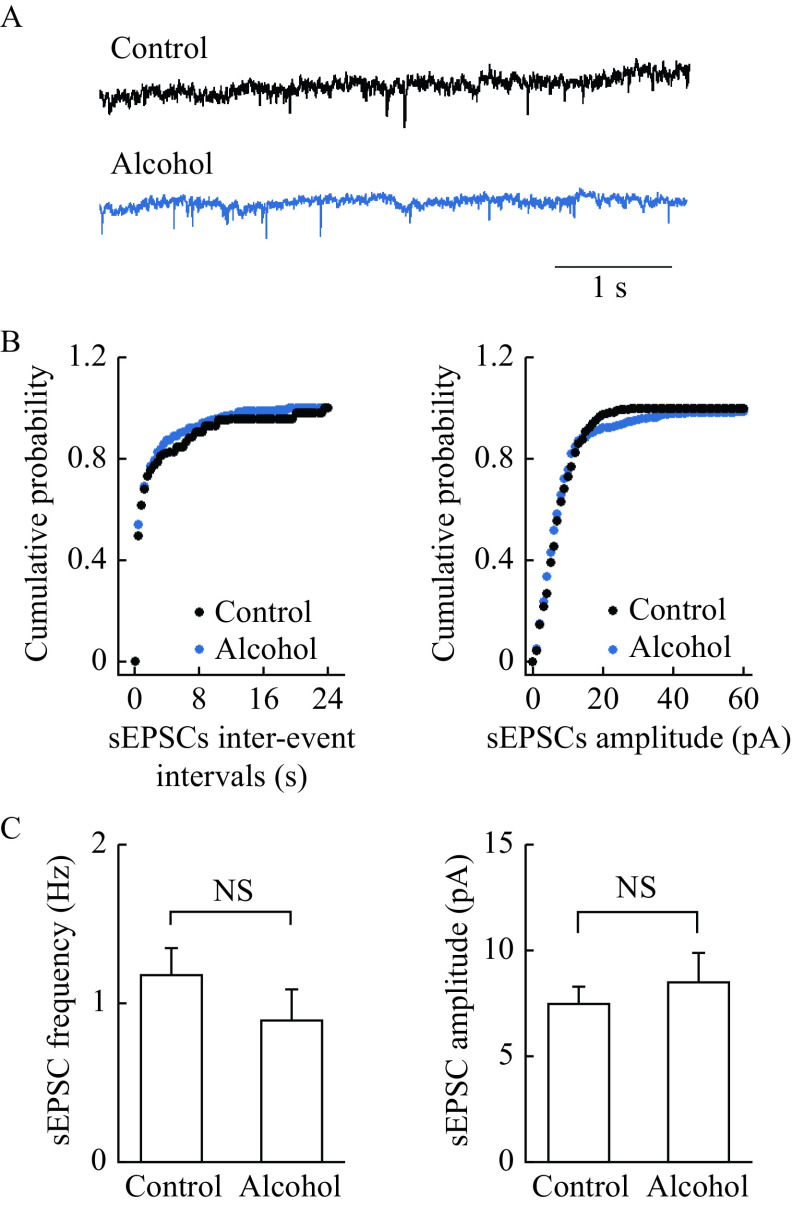
The frequency and amplitude of spontaneous excitatory postsynaptic currents did not differ between the control and alcohol groups.

Given that alcohol vapor exposure-induced withdrawal behaviors, the glutamatergic transmission may change accordingly. After testing behavioral changes, the animals were sacrificed for electrophysiological experiments (***Fig. 5A***). We focused on the mPFC because this brain area is involved in anxiety and depression. Compared to the control group, the input-output relationship between α-amino-3-hydroxy-5-methyl-4-isoxazole-propionic acid (AMPA)-mediated responses and paired-pulse ratio (PPR) did not change in the alcohol group (***Fig. 5B*** and ***C***). We found that the NMDA/AMPA ratio increased in the alcohol group, compared to the control group (***Fig. 5D***). Furthermore, we found that neither the frequency nor amplitudes of spontaneous excitatory postsynaptic currents (EPSCs) of the alcohol model group changed compared with the control group (***Fig. 6A***–***C***). Taken together, it is suggested that the alcohol vapor exposure mice model with this novel device excessively increased NMDAR-mediated glutamatergic transmissions but not AMPAR-mediated responses.

## Discussion

In this study, we independently designed a device for establishing an animal model of chronic alcohol exposure. We conducted experiments to explore suitable conditions for alcohol vapor modeling by measuring the BAC and neuronal injury in mice. Furthermore, this system induced motor coordination impairment, anxiolytic- and depression-like behavior. Accordingly, the NMDAR, but not AMPAR-mediated responses in mPFC changed, while the frequency and amplitude of sEPSCs between control and alcohol groups did not differ. These results demonstrated that our constructed vapor chamber system could successfully model excessive alcohol drinking and its related behaviors in mice, providing a method for researchers to study alcohol exposure-induced injury and disorders.

### Design and construct the alcohol vapor chamber

Accumulating studies showed alcohol vapor chamber is a better model for alcohol consumption in humans^[18–19]^. However, the quantity, frequency, and duration parameters are crucial for this system to make the animals that are susceptible to suffer from alcohol. Till now, there was no such system in China. Here, we designed and constructed an alcohol vapor chamber. Compared with relevant systems abroad, it has the following advantages: a) Optimized controller can adjust the large flow of alcohol vapor and shorten the animal modeling time. b) A multi-module function ensures the balance of oxygen and carbon dioxide concentration and reduces animal mortality. c) Environmental monitoring module monitors alcohol concentration in exposure chamber and maintaines a stable proportion of alcohol vapor. d) Large capacity exposure chamber combined with a multi-module can increase the number of modeling animals. e) Alcohol vapor progressively increases from low to high levels over time; this gradual increase in alcohol vapor allowes the animal to develop tolerance to alcohol without having any noticeable detrimental health effects. f) Chronic alcohol vapor inhalation allows the experimenter to control the dose, duration, and pattern of alcohol exposure. g) The pressure in the chamber is balanced with the atmospheric pressure by adjusting the compressor and vacuum pump, which does not cause negative health outcomes for mice.

### Evaluate the alcohol vapor chamber

We explored the relationship between the concentration of alcohol vapor used in modeling and the alcohol concentration. The system conditions are appropriate to make the BAC at high levels (1.5–2.0 mg/mL), including air flow rate, exposure frequency and duration, temperature, and air pump speed. We did not use an alcohol dehydrogenase inhibitor, which is more physiologically relevant. The additional advantage was that the exposure time is shorter than that in other reports. Therefore, the evaluation of this chamber suggested that it is sufficient to model the animals based on BAC. Given that the BAC can be stabilized in 1.5 to 2 mg/mL, the alcohol will enter the brain through the blood-brain barrier. Our findings demonstrated that the vapor exposure caused neural damage, which is another evaluation for this system^[20]^.

### Alcohol withdrawal caused anxiolytic-like and depression-like behavior as well as motor impairment in mice

Given that alcohol withdrawal eliciting negative consequences, anxiolytic- and depression-like behaviors will occur^[21]^. Therefore, our study used test batteries to evaluate these behaviors after the construction of the vapor chamber system, the mice were first put in a relatively invasive test, this would have the greatest impact on behavior in subsequent less invasive tests^[22]^, so we carried out the open field test, rod rotation experiment, and forced swim test successively to avoid the impact of test order as possible. Our results suggested that alcohol exposure-induced motor coordination impairment, depressive- and anxiety-like behaviors^[23–24]^. The three behavioral test results suggested that the animal model of chronic alcohol exposure was successfully established^[19]^. Recent studies have shown that alcohol self-administration reduced glutamatergic plasticity (increased NMDA/AMPA ratio)^[25]^. Acute alcohol suppressed NMDA activity, while chronic alcohol consumption enhanced NMDA function ^[26]^. It has shown that chronic alcohol exposure mediates the production of anxiolytic-like behaviors through the mPFC pathway^[27–28]^. We observed that alcohol increased the NMDA/AMPA ratio, but not the AMPAR-EPSCs in mPFC, suggesting that alcohol prevents NMDAR-mediated responses of mPFC neurons, which is consistent with reports that alcohol acts directly on NMDAR^[29]^. We verified whether alcohol alters the presynaptic transmission efficiency of the mPFC by recording PPR and sEPSCs, and the results suggested that presynaptic mechanism is not associated with alcohol exposure-induced behaviors.

### Conclusions and implications

This new modeling system has the advantages of short modeling time and a high success rate. It can be used in evaluating the effectiveness of drug intervention in alcohol-related behaviors. The method for this system was limited to passive vaporized ethanol exposure. The present findings cannot account for whether similar effects would be observed with voluntary consumption or being found only following physical dependence^[30]^. In the future, we plan to establish animal models of alcohol-induced complicated diseases including alcohol-induced hypertension, liver damage, and cerebrovascular diseases^[31]^.
